# Machine learning model for predicting age in healthy individuals using age-related gut microbes and urine metabolites

**DOI:** 10.1080/19490976.2023.2226915

**Published:** 2023-06-23

**Authors:** Seung-Ho Seo, Chang-Su Na, Seong-Eun Park, Eun-Ju Kim, Woo-Seok Kim, ChunKyun Park, Seungmi Oh, Yanghee You, Mee-Hyun Lee, Kwang-Moon Cho, Sun Jae Kwon, Tae Woong Whon, Seong Woon Roh, Hong-Seok Son

**Affiliations:** a Research & Development Team, Sonlab Inc, Seoul, Republic of Korea; b College of Korean Medicine, Dongshin University, Naju, Republic of Korea; c Department of Biotechnology, College of Life Sciences and Biotechnology, Korea University, Seoul, Republic of Korea; d Kyurim Korean Medical Clinic, Cheonan, Republic of Korea; e Department of Applied Statistics, Yonsei University, Seoul, Republic of Korea; f AccuGene Inc, Incheon, Republic of Korea; g Kimchi Functionality Research Group, World Institute of Kimchi, Gwangju, Republic of Korea; h Microbiome Research Institute, LISCure Biosciences Inc, Seongnam, Republic of Korea

**Keywords:** Age; prediction; urine, fecal, metabolomics, metataxonomics

## Abstract

Age-related gut microbes and urine metabolites were investigated in 568 healthy individuals using metataxonomics and metabolomics. The richness and evenness of the fecal microbiota significantly increased with age, and the abundance of 16 genera differed between the young and old groups. Additionally, 17 urine metabolites contributed to the differences between the young and old groups. Among the microbes that differed by age, *Bacteroides* and *Prevotella* 9 were confirmed to be correlated with some urine metabolites. The machine learning algorithm eXtreme gradient boosting (XGBoost) was shown to produce the best performing age predictors, with a mean absolute error of 5.48 years. The accuracy of the model improved to 4.93 years with the inclusion of urine metabolite data. This study shows that the gut microbiota and urine metabolic profiles can be used to predict the age of healthy individuals with relatively good accuracy.

## Introduction

Chronological age is a major risk factor for functional impairment, chronic disease, and mortality. The complex social and medical costs associated with biological aging are continually increasing and represent an ever-growing challenge.^1^ In keeping with the unprecedented growth rate of the global aging population, there is a need for a better understanding of the biological aging process and determinants of healthy aging.^2^ As aging is a complex trait influenced by individual genetic factors, diet, lifestyle, and environmental factors, it is very difficult to measure the degree of aging simply by age. However, developing tools to measure aging is an important challenge for predicting health. Therefore, several studies have been conducted to measure aging with various biological age predictors.^3–10^

The human gut contains a vast and diverse microbial ecosystem that has co-evolved with humans and is essential for human health.^11^ The risk or severity of numerous diseases and disorders in a host is associated with the gut microbiome, and accurate trait prediction based on microbiome characteristics is an important issue.^12^ During the past decade, accumulating evidence suggests that the gut microbiota affects body weight, energy homeostasis, innate immune system, and aging.^13,14^ Epidemiological studies have shown that loss of diversity in the core microbiota is associated with frailty.^15^ Urine is a valuable resource for the early detection of sensitive biomarkers because it can rapidly reflect changes in the body. Furthermore, as an analytical tool, urine has advantages over other biofluids, such as relatively less complex sample pretreatment, lower protein content, and sample complexity, including fewer intermolecular interactions.^16^ Lastly, urine is a more convenient sample to collect than blood because it can be taken non-invasively.^17^ Therefore, urine can provide fingerprints for personalized endogenous metabolite markers that can interpret age.^18^ Metabolic profiling of biofluids (such as urine, plasma, and fecal water) can be statistically integrated with microbial profile data using multivariate computational modeling.^19^

Machine learning can analyze large-scale data and combine them into predictions of disease risk, diagnosis, prognosis, and appropriate treatments.^20^ Recently, studies have been conducted to predict host characteristics by analyzing microbial and metabolic patterns using machine learning techniques.^21,22^ Predicting age using machine learning on multi-omics data can lead to better predictive models owing to interactions between features. A previous machine learning predictive model, a human microbiome clock, achieved an accuracy of 5.91 years mean absolute error in cross-validation.^23^ However, biological differences, such as race, country, culture, and lifestyle, can lead to prediction errors.^24^ Therefore, for industrial application, a population-based research design with biological characteristics similar to those of actual users is required.

In this study, age-related gut microbes and urine metabolites were identified in 568 healthy Koreans aged 20–80 years using a metataxonomics and metabolomics approach. In addition, urine metabolites associated with aging-related gut microbes were identified and an optimal age prediction model was built using machine learning technology.

## Results and discussion

### Gut microbial profiling by age

**Figure 1.**
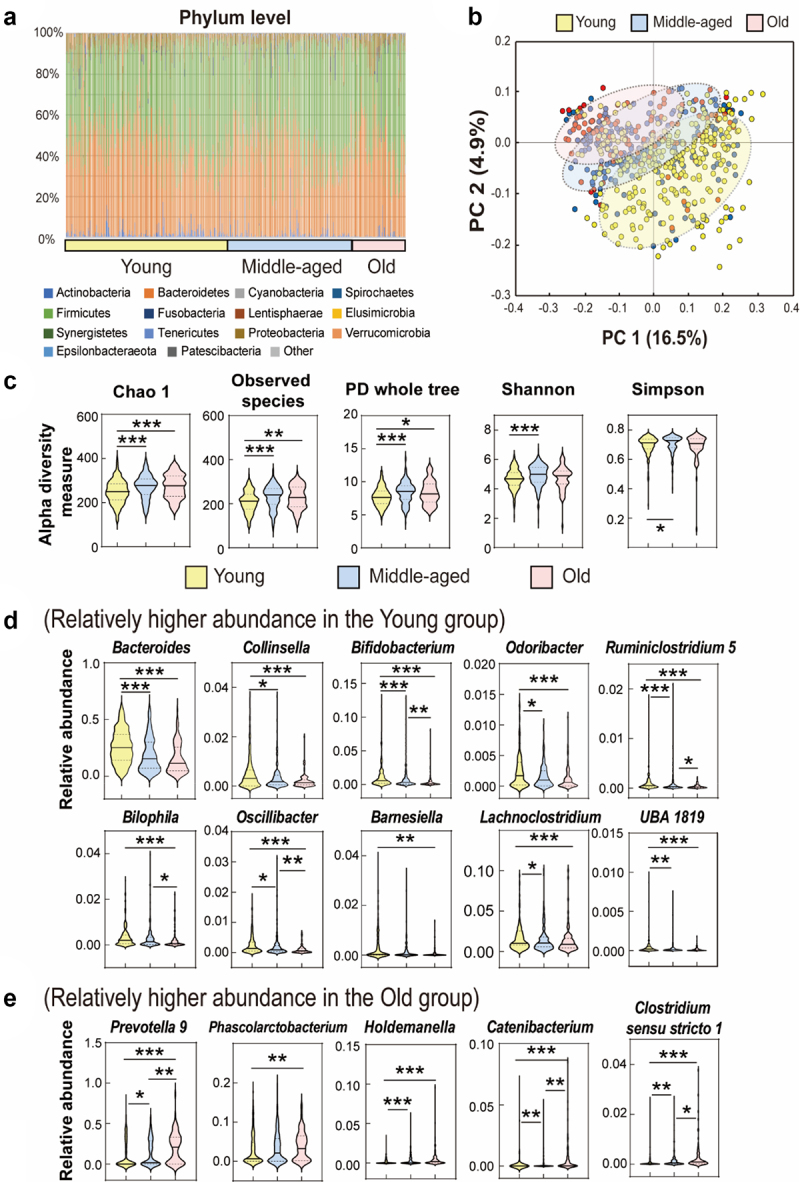
Analysis of gut microbial community profiles according to age. The samples were divided into three groups (young: 20–39 years, middle-aged: 40–59 years, and old: ≥60 years). (a) Relative abundance (%) of bacteria by ascending age groups at the phylum level. (b) Principal component analysis (PCA) of beta-diversity based on the operational taxonomic unit (OTU) level (unweighted UniFrac). (c) Alpha-diversities of microbial communities between young (yellow), middle-aged (blue), and old (red) groups (*p* value was calculated using a Kruskal–Wallis test; *p < .1, **p < .01, ***p < .001). (d, e) Violin plots of relative abundance of bacterial taxa (genus level) that contribute to differences in the linear discriminant analysis effect size (LEfSe) (LDA >2.0 and p < .05) between young and old groups. (d) Relatively higher abundance in the young group. (e) Relatively higher abundance in the old group. *p* value was calculated using a Kruskal–Wallis test; *p < .1, **p < .01, ***p < .001.

We performed 16S rRNA gene amplicon sequencing of 568 healthy individuals to investigate the pattern of gut microbes according to age. We divided the samples into three age groups based on their age distribution (young: 20–39 years, middle-aged: 40–59 years, and old: ≥60 years). The average relative abundances of Firmicutes and Bacteroidetes accounted for 89.29%, 90.40%, and 90.05% in the young, middle-aged, and old groups, respectively (Figure 1a). Recently, Leite et al.^25^ reported a positive relationship of the small intestinal abundance of phylum Proteobacteria with age. To validate whether the phylum Proteobacteria is an indicator taxon of the aging process, we categorized our cohort into young, middle, and old groups and surveyed the relative abundance of the phylum Proteobacteria. Although a significant difference in the relative abundance between the groups was observed, the phylum Proteobacteria did not increase with age in Korean subjects (Supplementary Figure S1). Given that an increased prevalence of the intestinal Proteobacteria is a potential diagnostic signature of dysbiosis and risk of disease,^26^ the irrelevance of Proteobacteria with age observed in this study might be attributed by i) our cohort comprising the specific disease-free healthy individuals and/or ii) compartmental difference (i.e., stool and small intestinal content) of microbial composition and structure.

Beta-diversity analysis based on the unweighted UniFrac index revealed that the distance between the young and middle-aged groups was smaller than the distance between the young and old groups (Figure 1b). However, the variance (PC1 = 16.5% and PC2 = 4.9%) in the principal component analysis was not high, indicating that the differences in microbial profiles between the groups were not significant. Alpha-diversity analysis using Chao1 showed considerable differences between the young and middle-aged groups and between the young and old groups (Figure 1c). In addition, the observed species and PD whole tree index results showed a pattern similar to that of Chao1. The Shannon index showed a significant difference only between the young and middle-aged groups. Although microbial diversity is generally known to decrease with age, conflicting results have been reported. Several studies have reported that microbial diversity continues to increase with age.^27,28^ For example, Yatsunenko et al.^24^ reported that gut bacterial diversity increases with age. Kong et al.^29^ showed that the gut microbial diversity increased in the longevity group (≥90 years old). The correlation between aging and microbial diversity may result from aging diseases.^29^ In other words, as many diseases increase with age,^30,31^ disease rather than age may be the direct cause of the decrease in microbial diversity. In the present study of healthy individuals, the microbial diversity increased with age. Therefore, to establish a relationship between age and microbial diversity, the health status of the participants should be considered.

Linear discriminant analysis (LDA) effect size (LEfSe) analysis revealed 28 taxa that differed between the young and old groups (LDA >2.0) (Supplementary Figure S2). Figure 1(d,e) shows violin plots showing the differences in the gut microbes of the young, middle-aged, and old groups at the genus level (LDA >2.0, *p* < .05). When the taxonomic composition was compared between the young and old groups, the abundance of *Clostridium* sensu stricto 1, *Holdemanella*, *Catenibacterium*, *Phascolarctobacterium*, and *Prevotella* 9 was significantly higher in the old group than in the young group. In contrast, the abundance of *Bacteroides*, *Barnesiella*, *Odoribacter*, *Collinsella*, *Bifidobacterium*, *Lachnoclostridium*, *Oscillibacter*, *Ruminiclostridium* 5, *UBA1819* (Family Ruminococcaceae), and *Bilophila* was lower in the old group than in the young group. In addition to age, sex influences the composition of the gut microbiome.^32^ The results of the age-related gut microbial profile analysis in males and females are shown in Supplementary Figures S3 and S4, respectively. In the age group comparison, although the relative abundance of some microbes differed between males and females, most microbes showed similar patterns regardless of sex.

In this study, the young group had a higher relative abundance of *Bacteroides* but a lower relative abundance of *Prevotella* 9. The role of *Bacteroides* in aging has been inconsistent in literature. Wilmanski et al.^33^ reported that a decrease in *Bacteroides* is a key feature of healthy aging. Conversely, Claesson et al.^34^ reported an increased predominance of *Bacteroides* in the elderly group (≥65 years) compared to that in the young group. Similarly, the exact biological trends of *Prevotella* 9 associated with aging have not yet been established. According to Wei et al.,^35^ the relative abundance of *Prevotella* 9 decreases with age, whereas the present study showed the opposite result. The contradictory results regarding the association of *Prevotella* 9 or *Bacteroides* with aging may be attributed to differences in dietary culture according to age. Gut microbial communities of individuals from developing countries were reported to be dominated by *Prevotella*,^36^ whereas those from developed countries were highly abundant in *Bacteroides*.^24^ Recently, the Korean diet has rapidly become westernized.^37^ Specifically, young people prefer animal-derived foods, whereas middle-aged and older people prefer grain- and plant-based foods (Supplementary Table S1). Therefore, more precise studies are needed to verify the correlation between age-specific eating habits of Koreans and *Bacteroides* or *Prevotella* 9.

Our findings confirmed a high prevalence of *Bifidobacterium* in the young group. *Bifidobacterium* have been identified as almost ubiquitous inhabitants of the human host, playing an important role in the gut from birth.^38^ *Bifidobacterium* plays an important role in the barrier effect and stimulation of the immune system associated with a range of beneficial health effects.^39^ Several studies using different techniques have repeatedly observed a trend toward decreasing bifidobacteria in the elderly population. Although this decline was associated with a reduction in adhesion to the intestinal mucosa, whether this is due to changes in the microbiota or in the mucus structure remains unclear.^40^ In addition, the extended use of antibiotics in the older population undoubtedly has a huge impact on the gut microbiota composition, decreasing the bifidobacterial population.^41^

### Metabolic profiling of urine by age

**Figure 2.**
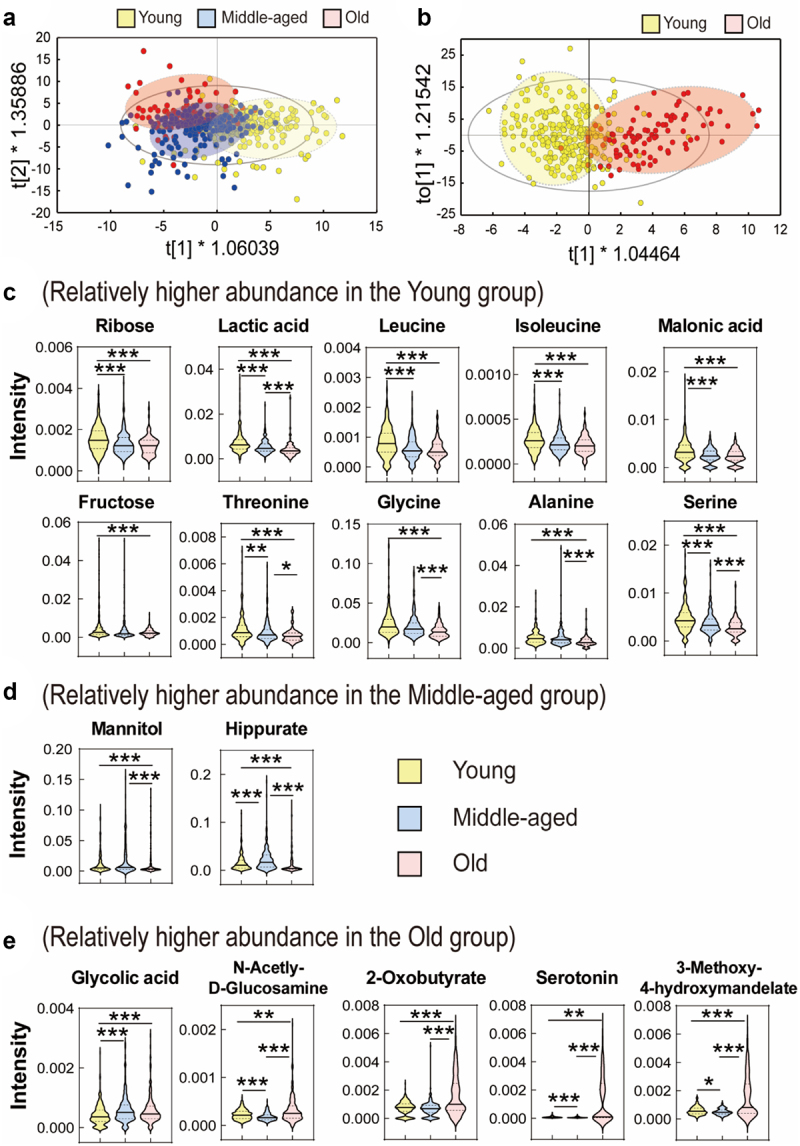
Orthogonal partial least squares discriminant analysis (OPLS-DA) score plot obtained from gas chromatography mass spectrometry (GC-MS) data of urine. The samples were divided into three groups (young: 20–39 years, middle-aged: 40–59 years, and old: ≥60 years). (a) OPLS-DA score plot of young (yellow), middle-aged (blue), and old (red) groups. (b) OPLS-DA score plot of young (yellow) and old (red) groups. Cross validation was performed using a permutation test that was repeated 200 times. No over-fitting was observed. (c-e) Violin plots of identified metabolites that contribute to differentiation between the young and old groups in the OPLS-DA model (VIP >1.0 and p < .05). (c) Relatively higher intensity in the young group. (d) Relatively higher intensity in the middle-aged group. (e) Relatively higher intensity in the old group. *p* value was calculated using a Kruskal–Wallis test; *p < .1, **p < .01, ***p < .001.

Metabolic differences in urine metabolites according to age were investigated using orthogonal partial least squares discriminant analysis (OPLS-DA). In the score plot, the young and old groups showed a tendency to separate, but the middle-aged group was distributed between the young and old groups (Figure 2a). Next, OPLS-DA was performed to identify metabolites that differed only between the young and old groups. The OPLS-DA score plot showed that the young and old groups were separated (Figure 2b). The metabolites responsible for discrimination are shown in Figure 2(c–e) (VIP >1.0, *p* < .05). Urine samples from the young group had higher levels of ribose, lactic acid, leucine, isoleucine, malonic acid, serine, fructose, threonine, glycine, and alanine and lower levels of glycolic acid, N-acetyl-D-glucosamine, serotonin, 3-methoxy-4-hydroxymandelate, and 2-oxobutyrate. The levels of mannitol and hippurate were the highest in the middle-aged group. To investigate sex-specific characteristics, the results of age-related metabolite analysis in males and females are shown in Supplementary Figures S5 and S6, respectively. In the age group comparison, although the content of some metabolites differed between males and females, most metabolites showed similar patterns regardless of sex.

A consistent underlying index of aging is a decline in cellular levels of the tripeptide glutathione.^42^ Glutathione is an essential thiol antioxidant produced in the cytosol of all cells that plays a key role in protection against oxidative stress by neutralizing free radicals and reactive oxygen species.^42^ The precursors of glutathione include cysteine, glutamate, and glycine. In the present study, glycine levels decreased with increasing age. The association between glycine and age may also be associated with the mitochondria. Hashizume et al.^43^ reported that defects in glycine metabolism in mitochondria are in part responsible for the reduction in mitochondrial translation, and consequently lead to age-related respiratory defects. They also confirmed that glycine supplementation restored respiratory defects in elderly fibroblasts.^43^ Serine metabolism is involved in cellular antioxidant functions. Serine supplementation increases Sirt1 protein deacetylase expression, decreases reactive oxygen species levels in the hypothalamus, and decreases pro-inflammatory cytokine levels.^44^ Duan et al.^45^ reported that impairment of glycine, serine, and threonine metabolism induces aging.

Hippurate is a glycine conjugate of benzoic acid that is associated with the gut microbiome^46^ and kidney function.^47^ Previous animal and human studies have reported an association between hippurate use and age. In a study by Williams et al.,^48^ a relatively low and variable amount of hippurate was observed in the urine of 4- and 6-week-old animals, but the levels increased and were less variable with increasing age. They argued that hippurate-like directional changes in urine are associated with maturation of the gut microbiota and the development of kidney function with age.^48^ An age-dependent increase in urine hippurate concentration was also confirmed in an aging study in dogs.^49^ Likewise, in the studies by Siqueira et al.^50^ and Wijeyesekera et al.,^51^ age and hippurate concentration were positively correlated. However, a study by Psihogios et al.^52^ showed lower concentrations of hippurate in participants aged >50 years than in those aged <35 years. In the present study, the hippurate levels in the middle-aged group were higher than those in the young group, but the hippurate level of the old group was lower than that of the middle-aged group. As the levels of hippurate are significantly affected by diet, disease, and exposure to certain toxins,^46^ a definitive interpretation is difficult. However, since many existing studies focus on urine hippurate as one of the biomarkers of aging, a research design that considers various variables such as race, disease, and diet is necessary for a clear interpretation.

### Integration of urine metabolites and fecal microbes

**Figure 3.**
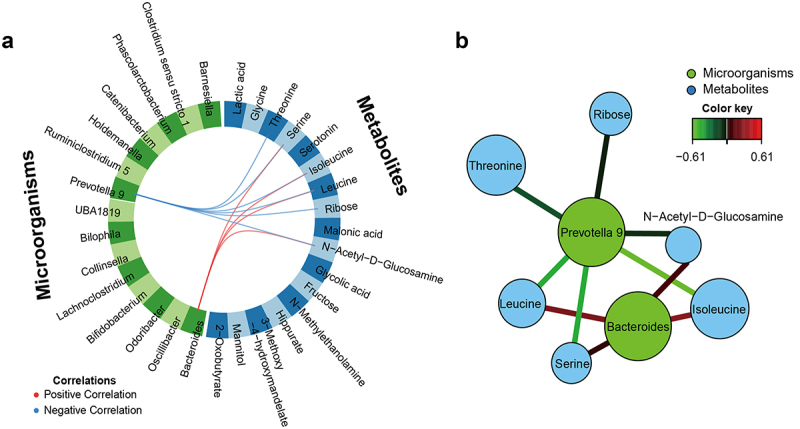
Data integration analysis for biomarker discovery using latent components (DIABLO) graphical outputs on the Korean age study. (a) Circos plot of correlations between urine metabolites and fecal microorganisms. Each dataset is assigned a different color: metabolites are in blue blocks and microorganisms in green blocks. Red and blue lines indicate positive and negative correlations between two variables, respectively (*r* ≥ |0.50|). (b) Network visualization of urine metabolites and fecal microorganisms derived from circos plots. Each dataset is assigned a different color: metabolites are in blue circles and microorganisms in green circles. Red and green lines indicate positive and negative correlations between two variables, respectively.

Graphical outputs from data integration analysis for biomarker discovery using the latent components (DIABLO) showed significant positive or negative correlations between urine metabolites and fecal microbes (Figure 3a). Among the microbes that differed by age, *Bacteroides* and *Prevotella* 9 were confirmed to be correlated with some urine metabolites. Figure 3(b) shows the network analysis results derived from the circos plot. The relative abundance of *Bacteroides*, with high levels in the young group, was positively correlated with the levels of serine, leucine, isoleucine, and N-acetyl-D-glucosamine. Conversely, the relative abundance of *Prevotella* 9, which was high in the old group, was negatively correlated with the levels of threonine, serine, leucine, isoleucine, ribose, and N-acetyl-D-glucosamine.

Several studies have investigated the relationship between the gut microbiota and urine metabolites in diseases such as diabetes,^53^ cardiovascular disease,^54^ and depression.^55^ Some metabolites produced by gut bacteria can enter the host bloodstream through the intestinal circulation.^56^ Wikoff et al.^57^ reported that one-third of the small molecules in the bloodstream may originate from gut bacteria. In the present study, *Prevotella* 9 and *Bacteroides* showed negative and positive correlations, respectively, with urine metabolites. Both microorganisms are related to human eating habits. However, as there are few studies on this aspect and further studies are needed to establish a clear association between gut microbes and urine metabolites.

### Age prediction using machine learning

**Figure 4.**
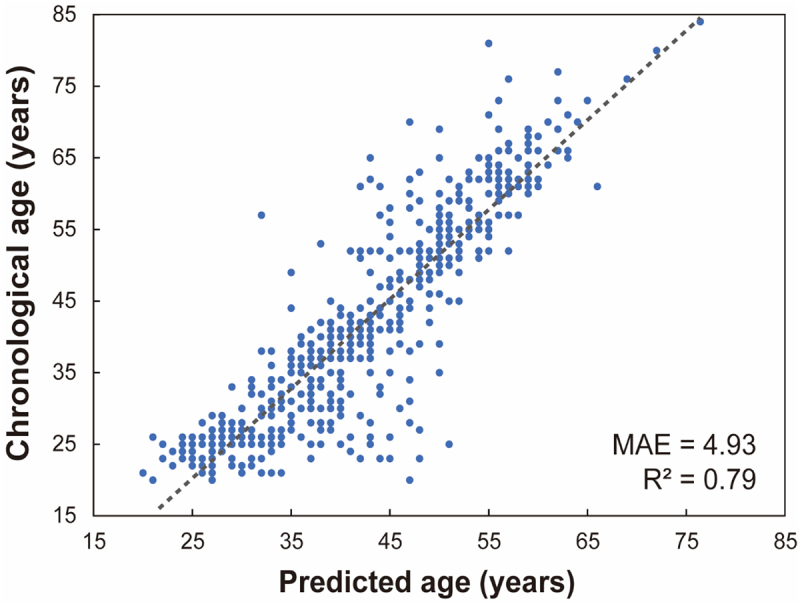
Linear regression scatter plot (Pearson correlation) of predicted age versus actual age based on gut microorganisms and urine metabolites (R^2^ = 0.79, *p* < .001). The x-axis shows the predicted age of the volunteers in years. The y-axis shows the actual age of the volunteers in years. Every blue dot displays one sample. The dotted line shows the linear correlation. R^2^ = coefficient of determination, MAE = mean absolute error in years.

For machine learning modeling, 379 urine metabolite features and 269 fecal gut microbiota features were used. We applied four machine learning algorithms to this dataset: gradient boosting (GB), eXtreme gradient boosting (XGBoost), light gradient boosting machine (LightGBM), and random forest (RF), to determine the optimal age prediction model. Supplementary Table S2 shows the overall mean absolute error of the different machine learning models. The machine learning algorithm XGBoost was shown to produce the best performing age predictors, with mean absolute error of 5.48 years. The accuracy of the model improved to 4.93 years with the inclusion of urine metabolite data. Figure 4 shows the results of a linear regression scatter plot between the age predicted by the XGBoost model and actual age (R^2^ = 0.79, *p* < .001). Various feature importance metrics can shed light on the overall importance of features. Based on the XGBoost model, each variable was ranked according to feature importance (Supplementary Table S3).

Using machine learning on multi-omics data to predict age can uncover new predictive models and biomarkers owing to interactions between features. This could be because of cross-interactions that occur when the value of one variable depends on another variable. In this case, even if a single variable does not seem to be significant for aging, the interaction term can contribute to predicting age. In this study, because the interaction of Actinobacteria and Bacteroidetes in machine learning contributed the most to improving the model performance, it is likely that it is the most important variable in age prediction. Another advantage is that machine learning methods are suitable for identifying biomarkers in variables with linear relationships. Microbiome and metabolite analyses require clear grouping for biomarker discovery. This study established three groups (young, middle-aged, and old) to identify age-related biomarkers and performed microbial community and metabolite analyses. However, this method is not suitable for variables with linear relationships. For example, the age variable was set according to the subjective opinion of the researcher in the group setting, because there was no clear distinguishing indicator. In this case, close variables (e.g., 39 years in the young group and 40 years in the middle-aged group) could cause errors in the potential biomarker search. In contrast, machine learning recognizes each linear variable as a separate variable and enables a biomarker search.

The aim of this study was to identify gut microbes and urine metabolites related to aging and to build an optimal age prediction model using machine learning technology. Age-related gut microbes and urine metabolites were identified in 568 healthy individuals. Although there is no minimum number of samples required to build a machine learning model, larger sample sizes can lead to the development of more robust models. Therefore, the use of more data, including open data, can improve the model performance. However, to use open data, it is necessary to match the sample analysis conditions and data-processing methods. Another limitation of the study was the uneven sample group size and female-to-male ratio. Unequal sample sizes between groups may affect the results of the statistical analysis. In addition, because the relative proportions of some microbes or metabolites may differ between sexes with age, obtaining data from a similar female-to-male ratio is important for building a robust model. Although 15 microbial genera and 17 metabolites showed differences between the young and old groups, some results differed from those of previous studies. This may be due to the different patterns of changes in gut microbes or urine metabolites with aging due to biological differences such as race, country, culture, and lifestyle. Nevertheless, this study showed that the gut microbiota and urine metabolic profiles can be used to predict the age of healthy individuals with relatively good accuracy, suggesting that noninvasive samples such as urine and fecal can provide aging-related information.

## Materials and methods

### Sample collection

**Figure 5.**
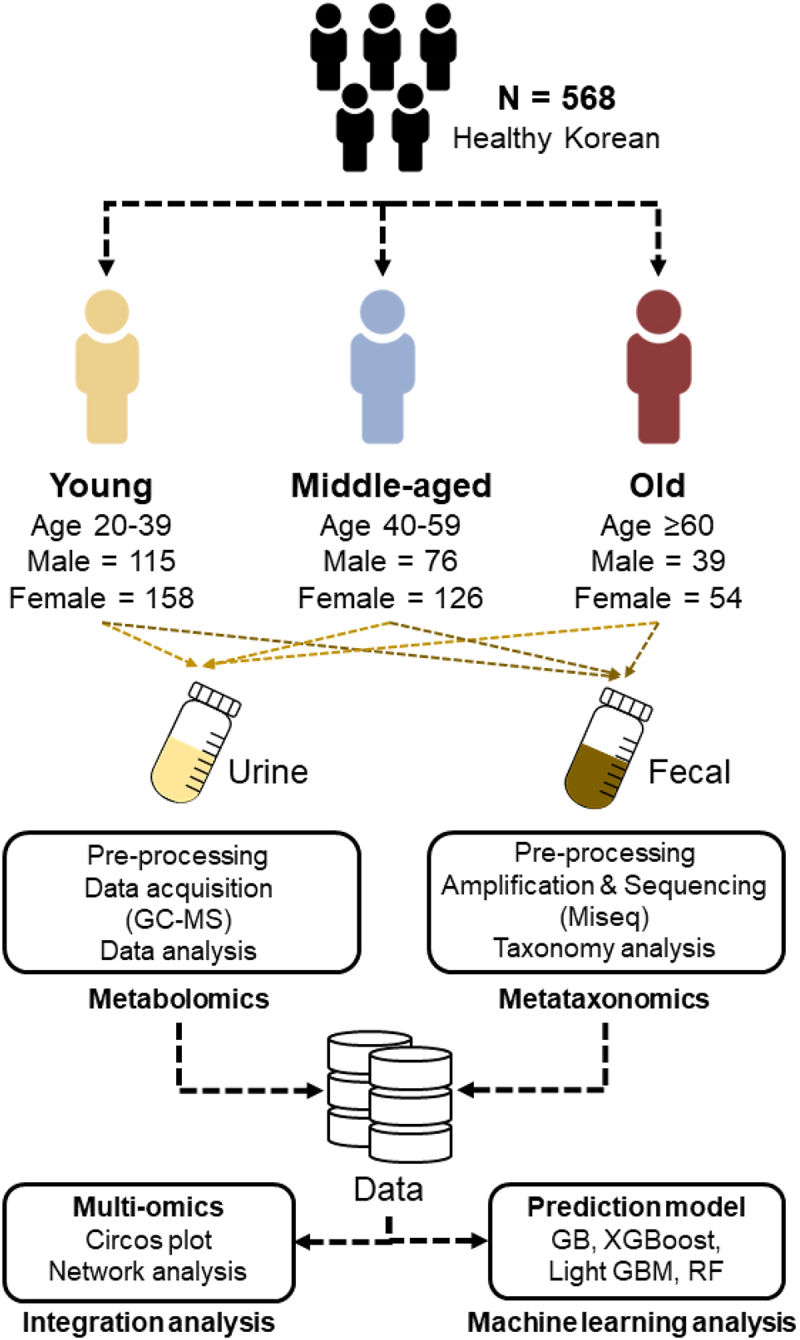
Sample characteristics and workflow of young, middle-aged, and old groups.

Stool and urine samples were collected from volunteers at the Naju Korean Medicine Hospital, Dongshin University (*n* = 568). The major exclusion criteria were as follows: (1) regular intake of medications, prebiotics or probiotics, and antibiotics within the past 3 months; (2) intestinal-related diseases; (3) other pathologies (type I or type II diabetes, cardiovascular or cerebrovascular diseases, cancer, neurodegenerative disease, rheumatoid arthritis, and allergies); and (4) pregnancy and lactation.^58^ The subjects fasted for 12 h before sample collection. Based on a previous study by Galkin et al.,^23^ we analyzed the samples by dividing them into three age groups according to age distribution (young: 20–39 years, middle-aged: 40–59 years, and old: ≥60 years) (Figure 5). General characteristics and anthropometric measurements of each age group are presented in Supplementary Table S4. Urine samples were collected on ice and stored at − 80°C until further analysis. Stool was collected using a stool sample collection kit (Fecal swab PLUS; Noble Biosciences Inc., Hwaseong, Korea). All stool samples were collected at the participants’ homes and immediately frozen (−20°C) in a freezer. It was then delivered to the laboratory within 24 h and stored in a − 80°C deep freezer. All samples were thawed in an ice bath before analysis. The study was conducted in accordance with relevant guidelines and regulations and the principles for human research. All the participants provided written informed consent. The medical ethics committee of Naju Korean Medicine Hospital of Dongshin University approved the study protocol (NJ-IRB-013).

### Microbiota analysis

#### DNA extraction and 16S rRNA gene sequencing

Fecal DNA was extracted using the AccuFAST automation system (AccuGene Inc., Incheon, Korea), according to the manufacturer’s instructions. For MiSeq sequencing, bacterial genomic DNA amplification was performed using primers containing 515fb and 806rb and Nextera adaptor sequences to target the V4 hypervariable region of the 16S rRNA genes.^59^ The 16S rRNA genes were amplified using 25 polymerase chain reaction (PCR) cycles and the KAPA HiFi HotStart ReadyMix (Roche Sequencing, Pleasanton, CA, USA). The PCR products (~250 bp) were purified using HiAccuBeads (AccuGene Inc., Incheon, Korea). Amplicon libraries were pooled at equimolar ratios. The pooled libraries were sequenced on an Illumina MiSeq system using the MiSeq Reagent Kit v2 for 500 cycles (Illumina, San Diego, CA, USA). The raw sequencing reads for all raw datasets were subjected to reference-based chimera filtering using VSEARCH v2.10.3.^60^ Chimeric filtered sequences were assigned to operational taxonomic units (OTUs) by OTU picking using the QIIME pipeline (http://www.qiime.org). Sequences were clustered using UCLUST into OTUs based on the SILVA 132 database (pre-clustered at 97% similarity threshold).

#### Data processing

To estimate the alpha diversity (within samples), we calculated the Chao1, observed species, PD whole tree, Shannon, and Simpson indices. Beta diversity (between samples) was computed using unweighted UniFrac to produce distance matrices, which were subsequently used for grouping samples into hierarchical cluster trees with the unweighted pair group method with arithmetic mean (UPGMA) and principal coordinate analysis representations (PCoA). LEfSe (http://huttenhower.sph.harvard.edu/galaxy/) analysis was performed using a Galaxy computational tool to estimate the effect size of each differentially abundant feature,^61^ with a cutoff value of the absolute log 10 LDA score > 2.0.

### Metabolic analysis

#### Sample pre-processing

Urine was aliquoted into 200 µL samples and prepared, including the depletion of urea with urease (40 units). Urine metabolites were extracted by adding 1 mL cold methanol (70% v/v) to 30 μL urine. The samples were vortexed and incubated at 37°C for 30 min, followed by centrifugation at 12,000 rpm for 5 min at 4°C to remove precipitated proteins. Quality control samples were prepared by pooling equal volumes (approximately 10 μL) of each sample before derivatization. Ten microliters of ribitol (0.5 mg/mL) was added as the internal standard. Finally, the collected supernatant from each sample was concentrated and dehydrated using an Eppendorf vacuum centrifuge for 3 h at 45°C.

#### Sample derivatization

After drying, 100 μL O-methoxyamine hydrochloride in pyridine solution (20 mg/mL) was added to each sample. After vortexing each sample for 30 s, all samples were incubated at 30°C for 90 min in the dark. Silylation was performed by adding 50 μL N-methyl-N-trimethylsilyl-trifluoroacetamide containing 1% trimethylchlorosilane. After vortexing each sample for 30 s, the samples were incubated at 37°C for 30 min. After centrifuging the samples at 13,000 rpm for 10 min, the supernatants were subjected to Gas chromatography – mass spectrometry (GC-MS) analysis. To measure the performance and stability of the system together with the reproducibility of the sample treatment procedure, quality control samples were analyzed every 30 samples throughout the run.

#### GC-MS analysis

The derivatized samples were analyzed using GC-MS (QP2020, Shimadzu, Kyoto, Japan). Rtx-5 MS with a fused silica capillary column (30 m × 0.25 mm ID, J&W Scientific, Folsom, CA, USA) was used for the separation of metabolites. The front inlet temperature was set to 230°C. The column temperature was isothermally held at 80°C for 2 min, increased by 15°C/min to 330°C, and isothermally held for 6 min. The transfer line and ion source temperatures were 250°C and 200°C, respectively. Ionization was achieved with a 70 eV electron beam. The flow rate of helium gas through the column was 1 mL/min. Twenty scans per second were recorded over the mass range of 85–500 m/z. Chromatograms and mass spectra were acquired using Shimadzu GC solution (Shimadzu, Kyoto, Japan).

#### Data processing

GC-MS data were converted from Shimadzu GC-MS Postrun Analysis software (Shimadzu, Kyoto, Japan) to the netCDF format file and processed using MetAlign software for peak detection and alignment.^62^ The resulting data (CSV-format file) were imported into AIoutput software for peak identification and prediction.^63^ Data normalization was performed using the internal standard and total ion current values. OPLS-DA of GC-MS data was performed to visualize the variance of metabolites using SIMCA-P 17.0 (Umetrics, Umea, Sweden). Cross-validation was performed using a permutation test repeated 200 times. Metabolites with VIP > 1.0 and *p* < 0.05 were considered metabolites that could discriminate groups. Metabolites were identified by comparing their mass spectra with those in AIoutput software, NIST 20.0 library, standard reagents (Supplementary Figure S8), and the human metabolome database (HMDB, http://www.hmdb.ca).

### Statistical analysis

Statistical analyses were performed using GraphPad Prism 9 (GraphPad Software, La Jolla, CA, USA). We evaluated the normality of the data using the D’Agostino-Pearson omnibus normality test. For parametric data, we conducted a Brown-Forsythe test to confirm equal variance, followed by either a one-way analysis of variance (ANOVA) or the Kruskal-Wallis test. To address multiple comparisons, we utilized the two-stage step-up method of Benjamini, Krieger, and Yekutieli. For non-parametric data, we used the Kruskal-Wallis test to compare groups, and for multiple comparisons, we employed Dunn’s post hoc test. We considered *p* values less than 0.05 to be statistically significant.

### Integration between microbes and metabolites

The R package mixOmics was employed for the integration of the two datasets (fecal taxonomic profile and urine metabolite data) using the DIABLO model.^64^

### Machine learning analysis

#### Feature extraction

AutoEncoder was employed to learn additional information from nonlinear combinations of high-dimensional features by training a compressed representation of the raw features. AutoEncoder comprises an encoder and a decoder, where the encoder transforms the input data into a compressed representation, and the decoder reconstructs the representation back to the input data.^65^ The encoder consists of four fully connected layers that contract 325 dimensions (as of the combined dataset) into 256, 128, 64, and 8 dimensional encoded vectors sequentially, whereas the decoder is a set of four fully connected layers that expand the encoded vectors into 64, 128, 256, and 325 dimensions. Each fully connected layer was followed by batch normalization,^66^ and ReLU and dropout^67^ layers.

#### Feature selection

Permutation feature importance scores feature importance by permuting a feature and tracking the effect of shuffling on overall accuracy.^68^ A total of 175 features (for the combined dataset) were utilized by specifying a subset of relevant features that exceeded the threshold using permutation feature importance scores.

#### Machine learning

Four different tree-based machine learning models: GB, XGBoost, LightGBM, and RF were applied to create age prediction models using metabolite and microbiome data.

## Supplementary Material

Supplemental Material

## Data Availability

The data presented in this study are openly available in MetaboLights repository at www.ebi.ac.uk/metabolights/MTBLS6313.
