# Supplementary material for: Machine learning model for predicting age in healthy individuals using age-related gut microbes and urine metabolites
Source: Gut Microbes. 2023 Jun 23;15(1):2226915. doi: 10.1080/19490976.2023.2226915 (PMC10291941; doi:10.1080/19490976.2023.2226915)
Supplement: Supplemental Material [file KGMI_A_2226915_SM3332.docx]

**Machine learning model for predicting age in healthy individuals using age-related gut microbes and urine metabolites**

Supplementary Material

**Supplementary Table 1.** Intake ratio of plant-based and animal-derived foods by age in Koreans (The seventh Korea National Health and Nutrition Examination Survey, 2020, Korea Disease Control and Prevention Agency).

|  | Age range (years) | | | |
| --- | --- | --- | --- | --- |
|  | 19-29 | 30-49 | 50-64 | 65≤ |
| Intake rate of plant -based food (%) | 73.73±0.81 | 77.24±0.43 | 78.54±0.46 | 81.55±0.48 |
| Intake rate of animal -derived food (%) | 26.27±0.81 | 22.76±0.43 | 21.46±0.46 | 18.45±0.48 |

**Supplementary Table 2.** Mean absolute error (years) of gradient boosting (GB), eXtreme gradient boosting (XGBoost), light gradient boosting machine (LightGBM), and random forest (RF) using gut microbiota and urine metabolites.

| Model | Feature extraction | Data source | | |
| --- | --- | --- | --- | --- |
|  |  | Gut microbiota | Urine metabolites | Gut microbiota + Urine metabolites |
| GB | O | 6.05 | 6.82 | 6.18 |
|  | X | 9.59 | 7.99 | 8.60 |
| XGBoost | O | 5.48 | 5.59 | 4.93 |
|  | X | 10.06 | 9.36 | 8.97 |
| LightGBM | O | 5.43 | 6.70 | 5.21 |
|  | X | 10.71 | 7.93 | 9.15 |
| RF | O | 6.04 | 7.41 | 6.74 |
|  | X | 10.46 | 8.55 | 10.04 |

**Supplementary Table 3.** Important features estimated by the eXtreme gradient boosting (XGBoost) model.

| **Rank** | **Features** | **Rank** | **Features** |
| --- | --- | --- | --- |
| **1** | Actinobacteria * Bacteroidetes | **11** | Quinic acid * Threonic acid |
| **2** | Allose * Isoleucine | **12** | Oxalate * Pyrogallol |
| **3** | Galacturonic acid * Oxalate | **13** | Glutamic acid * Isoleucine |
| **4** | Gallic acid * Uridine | **14** | 3-Methoxy-4-hydroxymandelate * Ribose |
| **5** | 1,6-Anhydroglucose * Histidine | **15** | Lactic acid * Pyrogallol |
| **6** | N-Formyl-L-Methionine * N-Methylethanolamine | **16** | Ascorbic acid * Histidine |
| **7** | Leucine * Maltose | **17** | Hippurate * Mannitol |
| **8** | Oxalate * Serotonin | **18** | homoserine * Uridine |
| **9** | Flavonifractor | **19** | b-Methylamino-L-alanine * Isoleucine |
| **10** | Galacturonic acid * Leucine | **20** | Actinobacteria |

The symbol * indicates a feature derived from the interaction between gut microbiota and urine metabolites

**Supplementary Table 4.** General characteristics and anthropometric measurements of each age group.

|  | | Young | Middle-aged | Old |
| --- | --- | --- | --- | --- |
| Age (years) | | 29.10 ± 5.30 | 49.50 ± 5.88 | 64.30 ± 4.01 |
| Age range (years) | | 20-39 | 40-59 | ≥60 |
| Sex | Male | 115 | 76 | 39 |
|  | Female | 158 | 126 | 54 |
| Weight (kg) | | 66.20 ± 14.00 | 64.21 ± 12.34 | 64.50 ± 8.95 |
| Height (m) | | 1.68 ± 0.08 | 1.63 ± 0.08 | 1.62 ± 0.07 |
| BMI (kg/m^2^) | | 23.34 ± 4.05 | 23.97 ± 3.51 | 24.558 ± 2.92 |


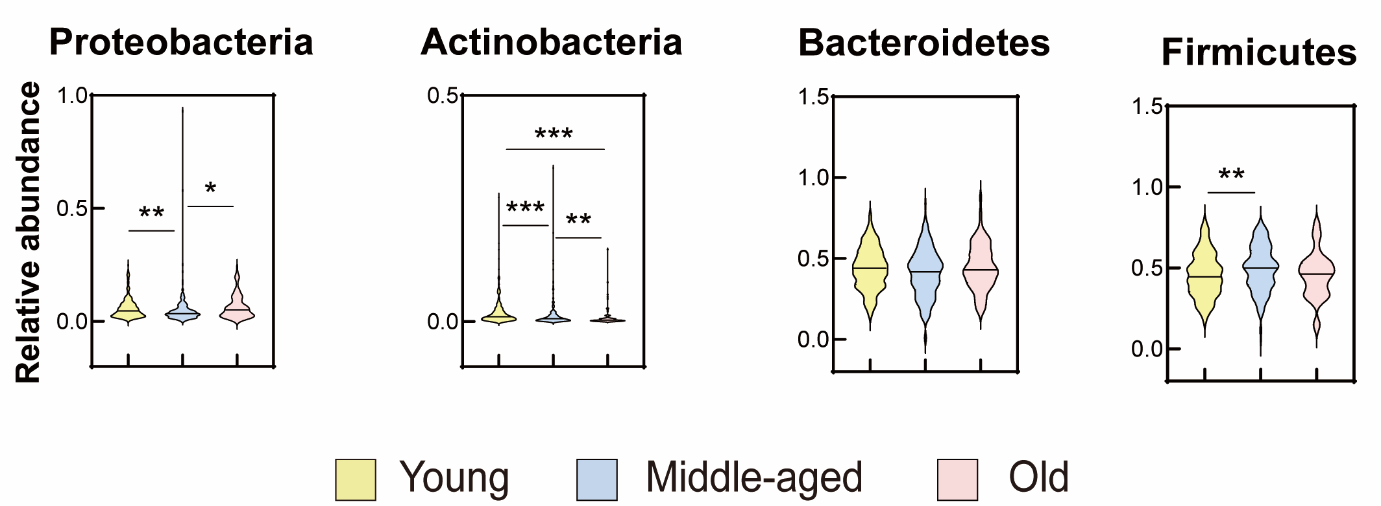


**Supplementary Figure 1.** Phylum-level abundance profiles of microbial communities between young (yellow), middle-aged (blue), and old (red) groups. (*p* value was calculated using a Kruskal–Wallis test; * *p* < 0.1, ** *p* < 0.01, *** *p* < 0.001).


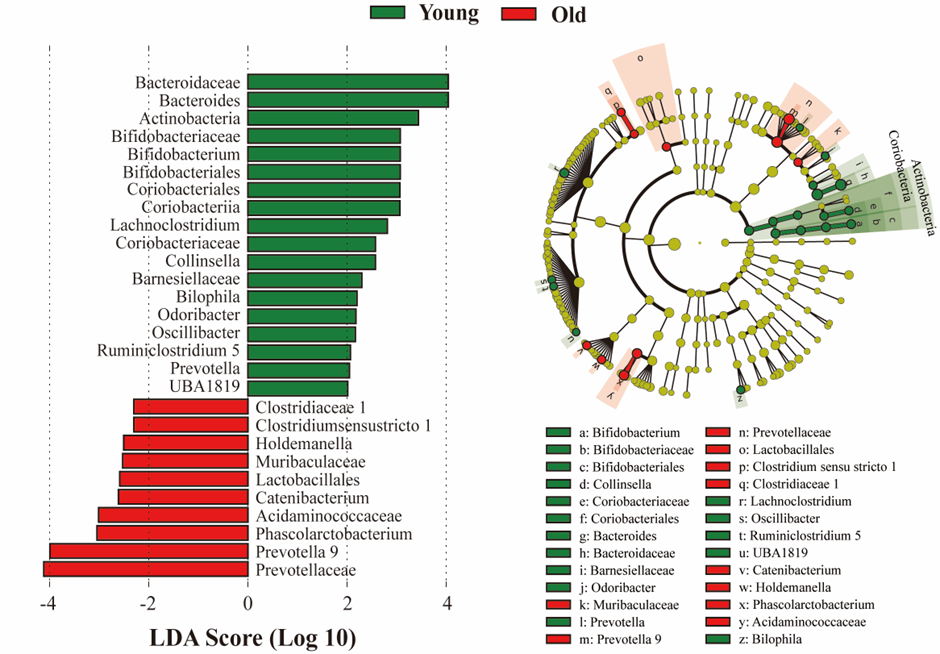


**Supplementary Figure 2.** Linear discriminant analysis (LDA) effect size (LEfSe) cladogram (LDA score > 2.0, *p* < 0.05) indicated differentially abundant taxa between young (green) and old groups (red).


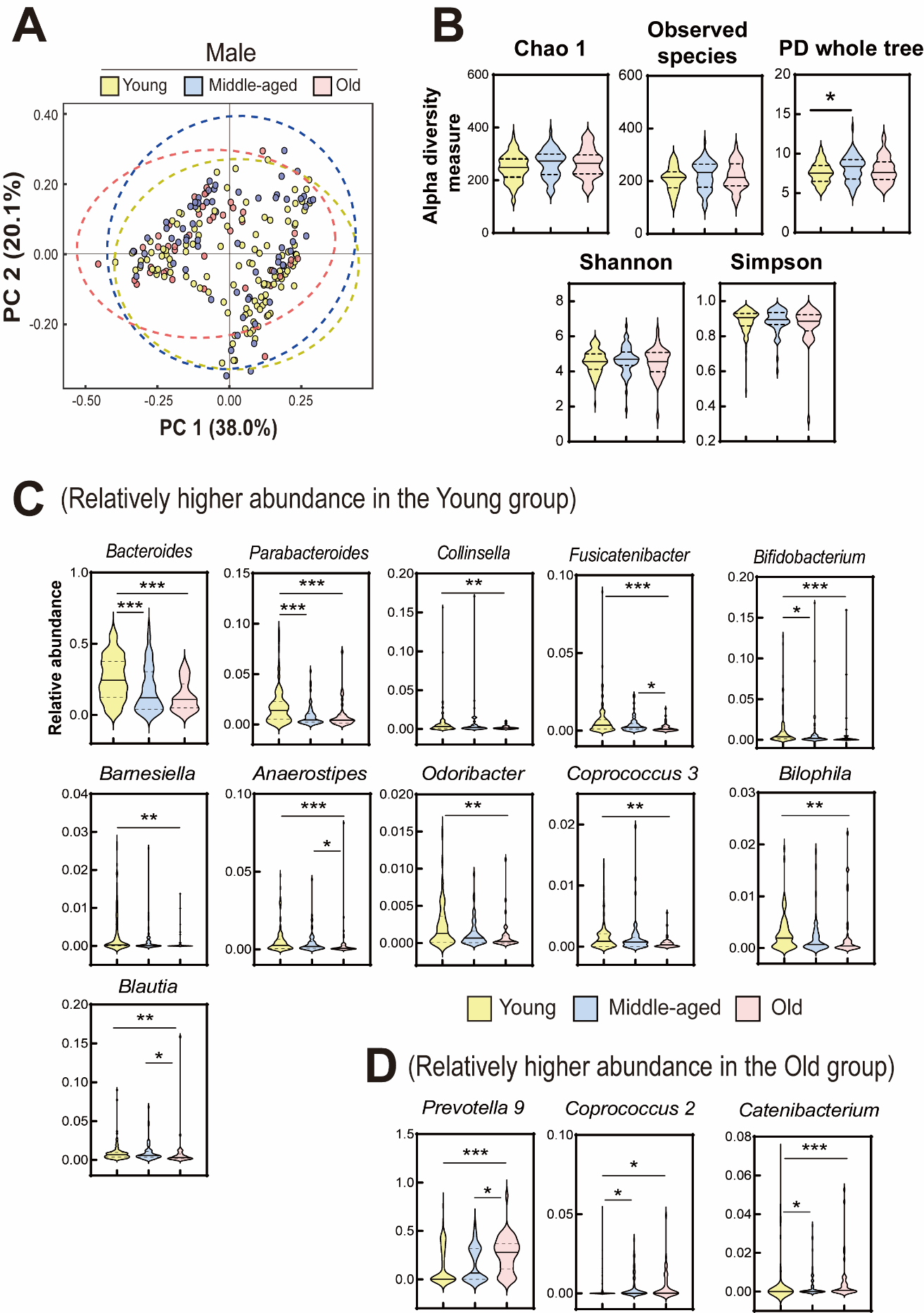


**Supplementary Figure 3.** Analysis of gut microbial community profiles according to age in male volunteers. The samples were divided into three groups (young: 20–39 years, middle-aged: 40–59 years, and old: ≥60 years). (A) Principal component analysis (PCA) of beta-diversity based on the operational taxonomic unit (OTU) level (unweighted UniFrac). (B) Alpha-diversities of microbial communities between young (yellow), middle-aged (blue), and old (red) groups. (C, D) Violin plots of relative abundance of bacterial taxa (genus level) that contribute to differences in the linear discriminant analysis effect size (LEfSe) (LDA > 2.0 and *p* < 0.05) between young and old groups. (C) Relatively high abundance in the young group. (D) Relatively high abundance in the old group. *p* value was calculated using a Kruskal–Wallis test; * *p* < 0.1, ** *p* < 0.01, *** *p* < 0.001.


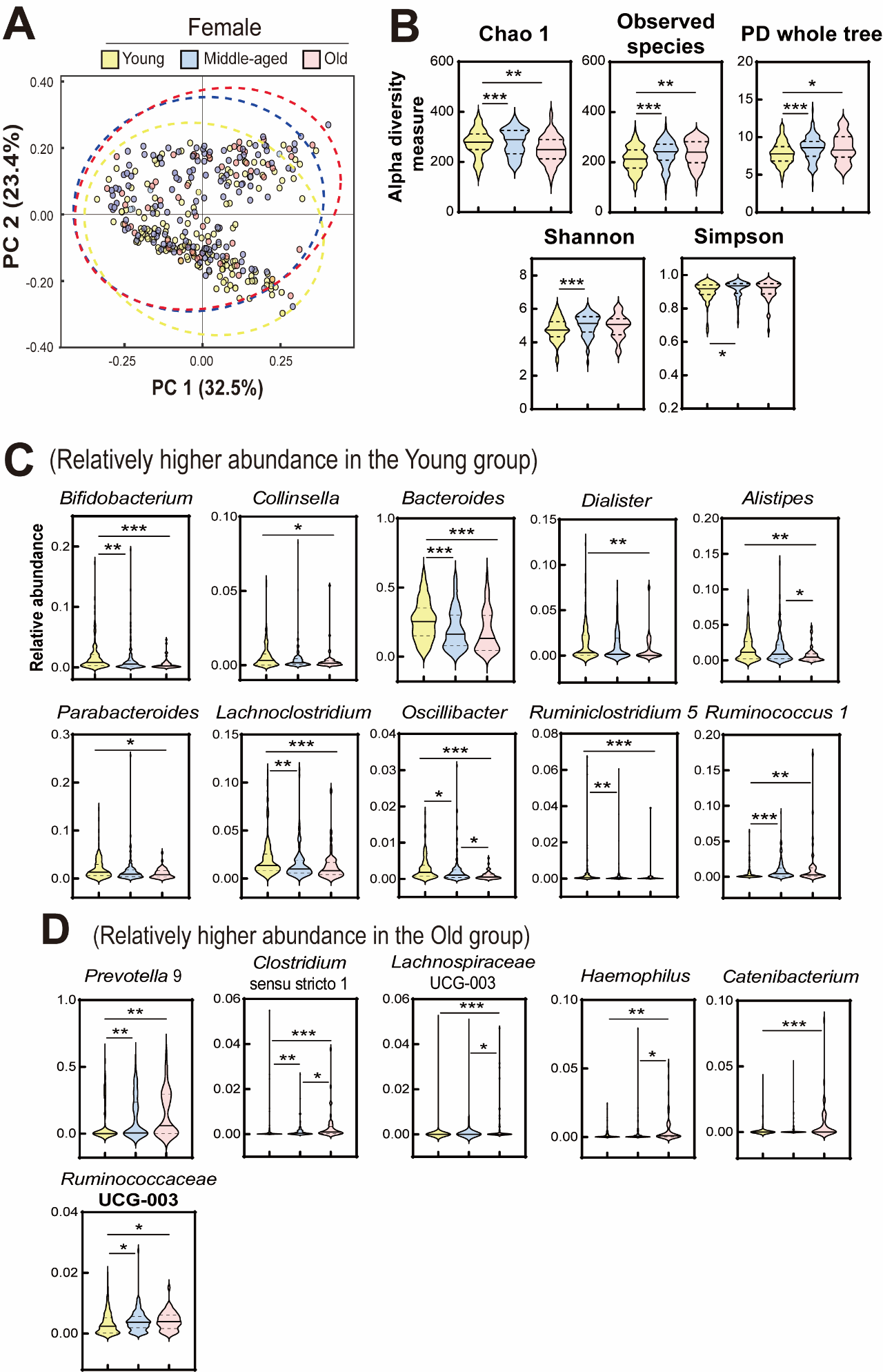


**Supplementary Figure 4.** Analysis of gut microbial community profiles according to age in female volunteers. The samples were divided into three groups (young: 20–39 years, middle-aged: 40–59 years, and old: ≥60 years). (A) Principal component analysis (PCA) of beta-diversity based on the operational taxonomic unit (OTU) level (unweighted UniFrac). (B) Alpha-diversities of microbial communities between young (yellow), middle-aged (blue), and old (red) groups. (C, D) Violin plots of relative abundance of bacterial taxa (genus level) that contribute to differences in the linear discriminant analysis effect size (LEfSe) (LDA > 2.0 and *p* < 0.05) between young and old groups. (C) Relatively high abundance in the young group. (D) Relatively high abundance in the old group. *p* value was calculated using a Kruskal–Wallis test; * *p* < 0.1, ** *p* < 0.01, *** *p* < 0.001.


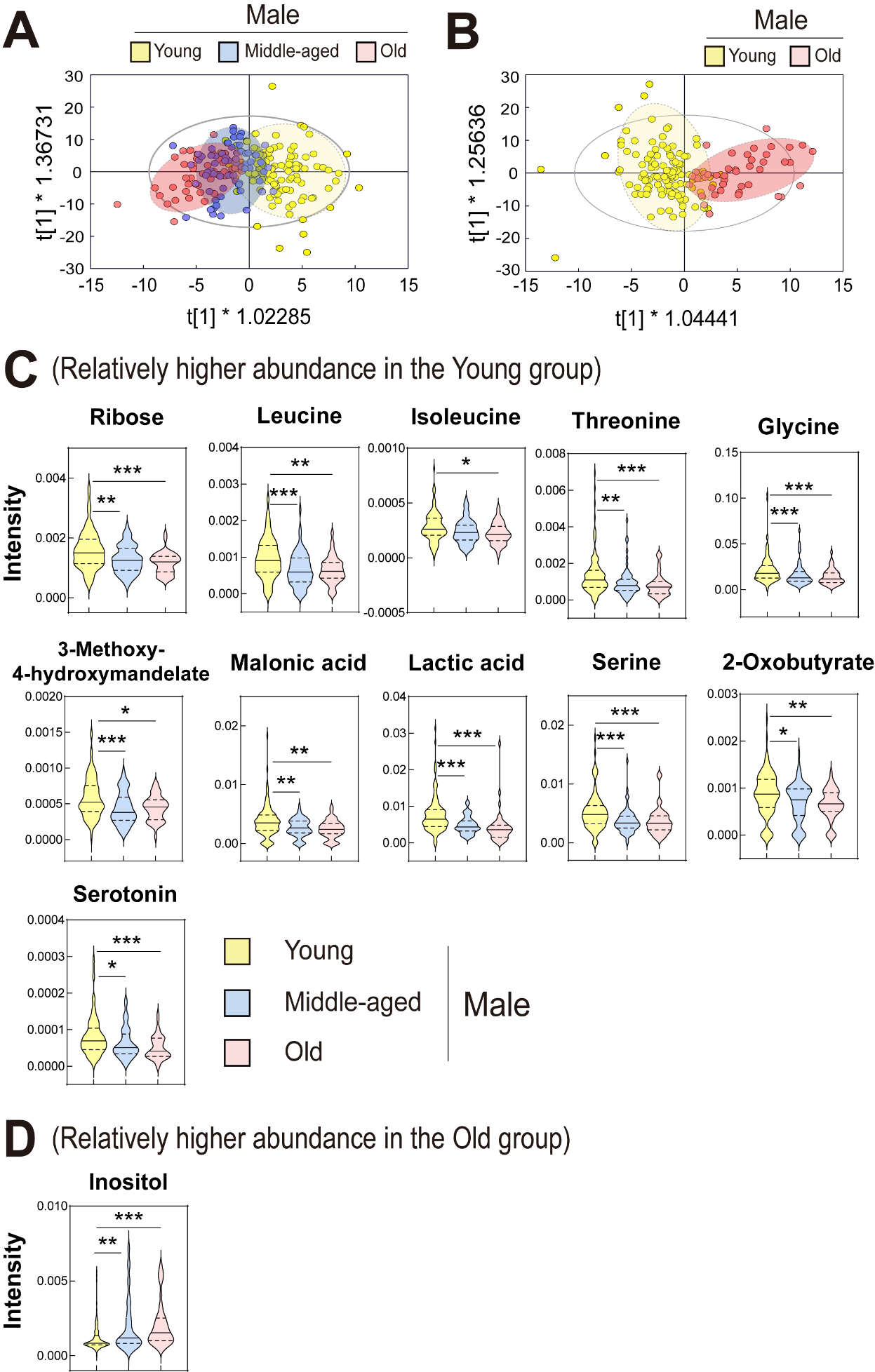


**Supplementary Figure 5.** Gas chromatography mass spectrometry (GC-MS)-based urine metabolite profiling by age group in male volunteers. The samples were divided into three groups (young: 20–39 years, middle-aged: 40–59 years, and old: ≥60 years). (A) Orthogonal partial least squares discriminant analysis (OPLS-DA) score plot of young (yellow), middle-aged (blue), and old (red) groups. (B) OPLS-DA score plot of young (yellow) and old (red) groups. Cross validation was performed using a permutation test that was repeated 200 times. No over-fitting was observed. (C, D) Violin plots of identified metabolites that contribute to differentiation between the young and old groups in the OPLS-DA model (VIP > 1.0 and *p* < 0.05). (C) Relatively higher abundance in the young group. (D) Relatively higher abundance in the old group. *p* value was calculated using a Kruskal–Wallis test; * *p* < 0.1, ** *p* < 0.01, *** *p* < 0.001.


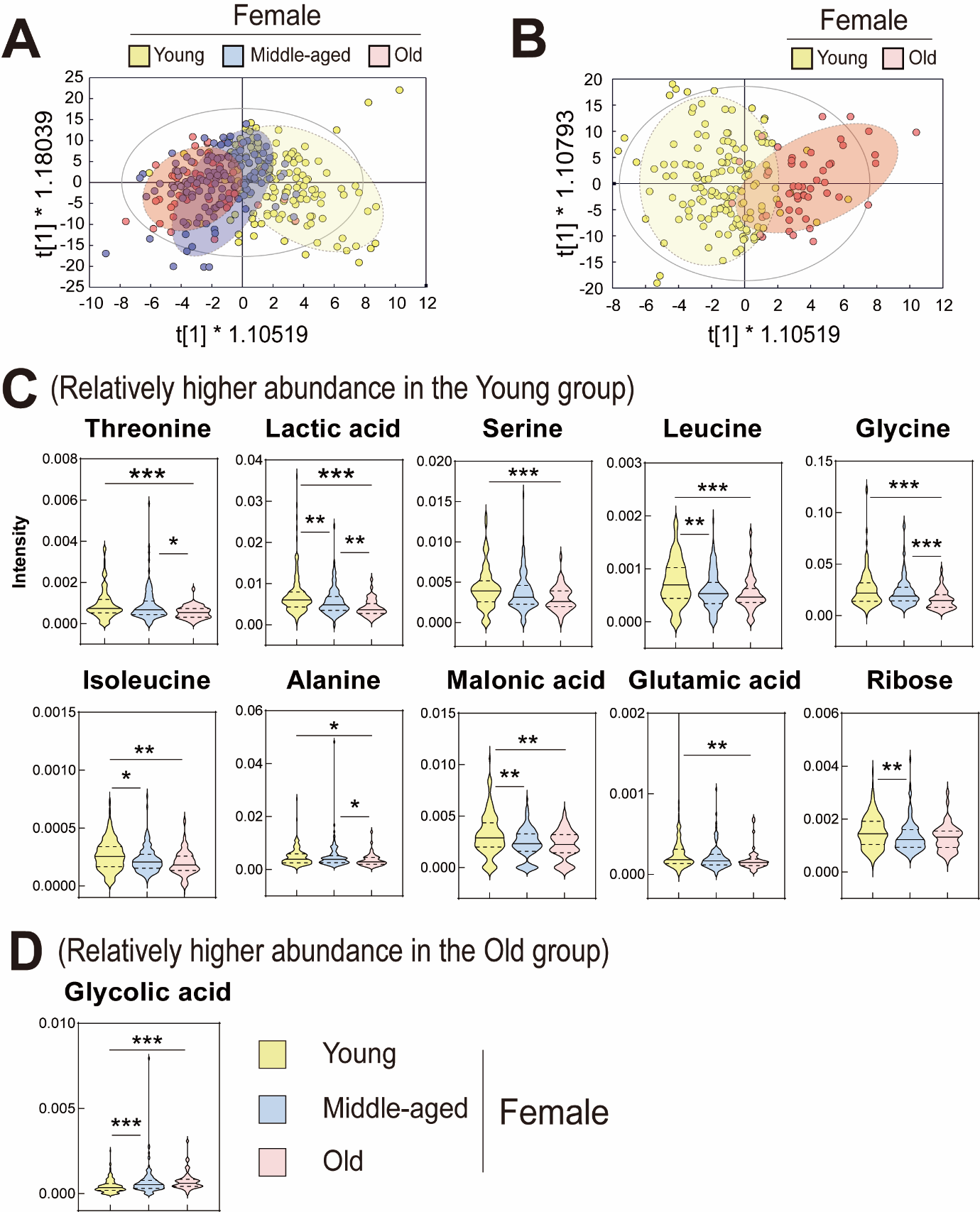


**Supplementary Figure 6.** Gas chromatography mass spectrometry (GC-MS) based urine metabolite profiling by age group in female volunteers. The samples were divided into three groups (young: 20–39 years, middle-aged: 40–59 years, and old: ≥60 years). (A) Orthogonal partial least squares discriminant analysis (OPLS-DA) score plot of young (yellow), middle-aged (blue), and old (red) groups. (B) OPLS-DA score plot of young (yellow) and old (red) groups. Cross validation was performed using a permutation test that was repeated 200 times. No over-fitting was observed. (C, D) Violin plots of identified metabolites that contribute to differentiation between the young and old groups in the OPLS-DA model (VIP > 1.0 and *p* < 0.05). (C) Relatively higher intensity in the young group. (D) Relatively higher intensity in the old group. *p* value was calculated using a Kruskal–Wallis test; * *p* < 0.1, ** *p* < 0.01, *** *p* < 0.001.


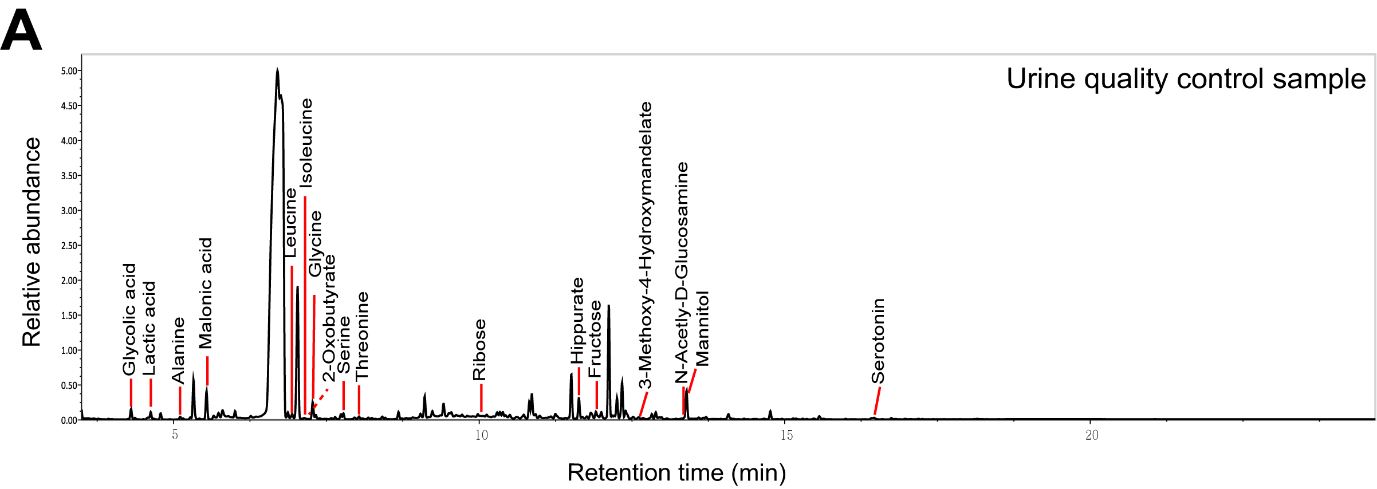


**Supplementary Figure 7.** Total ion chromatogram (TIC) of metabolites extracted from human urine quality control sample.


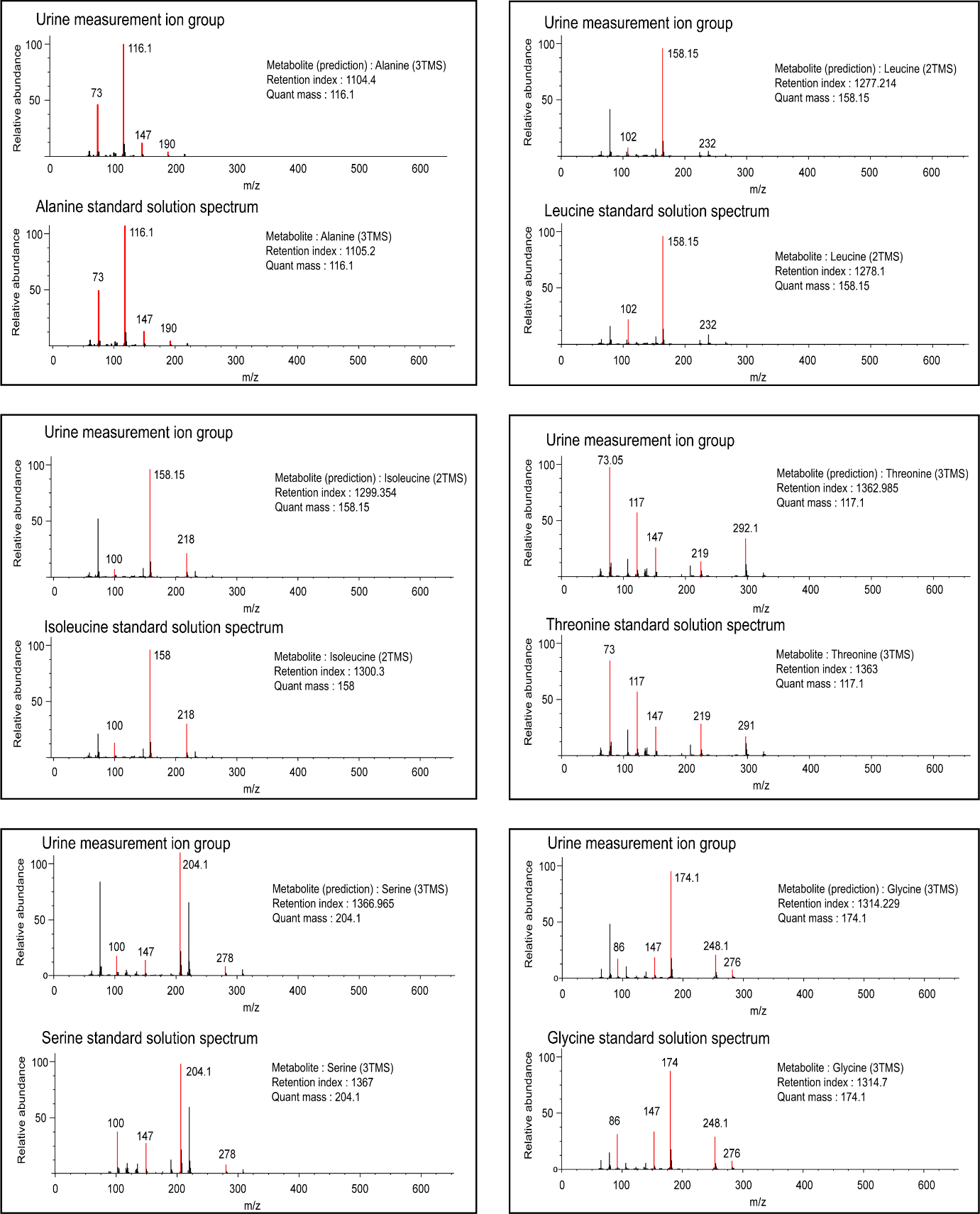


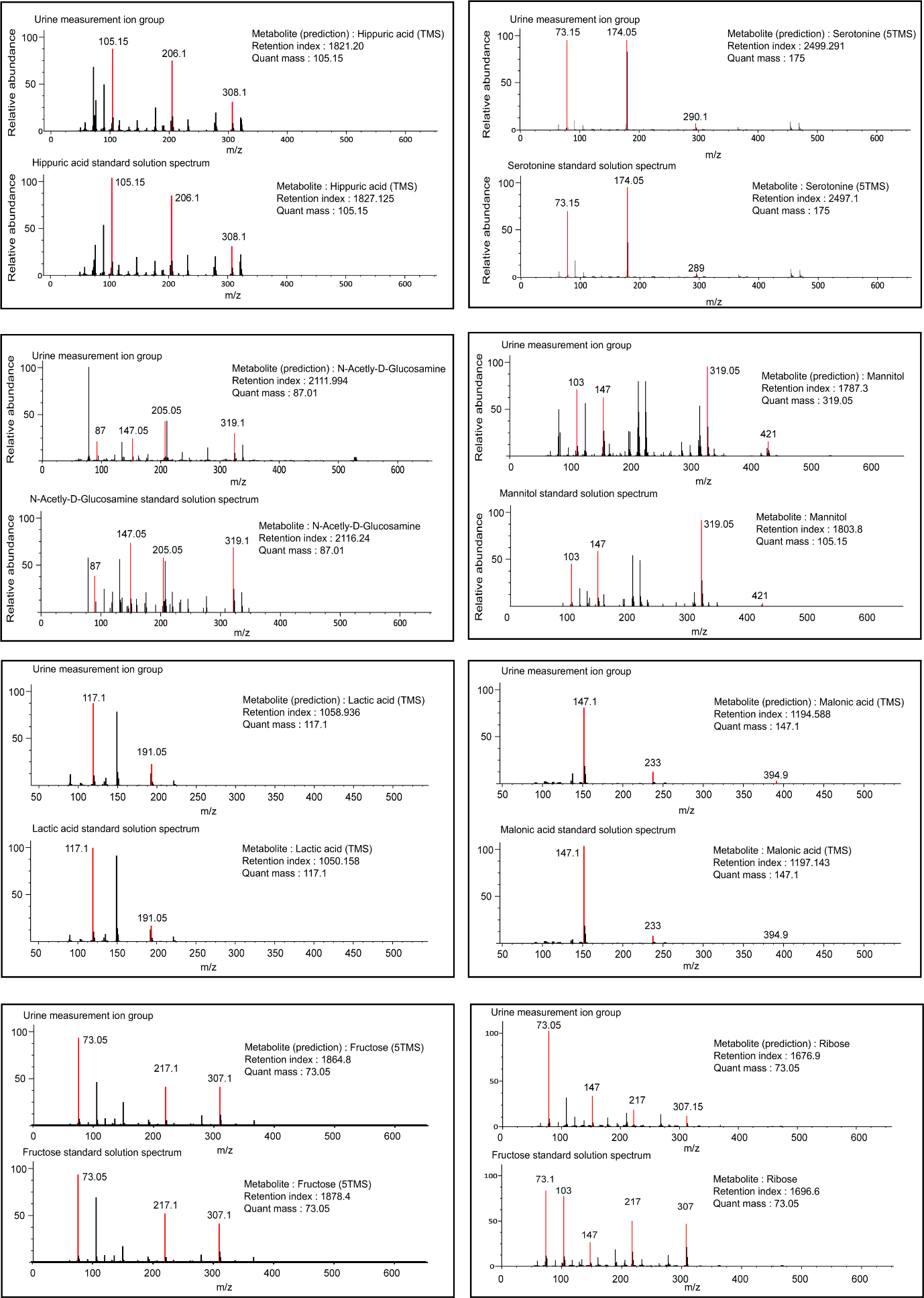


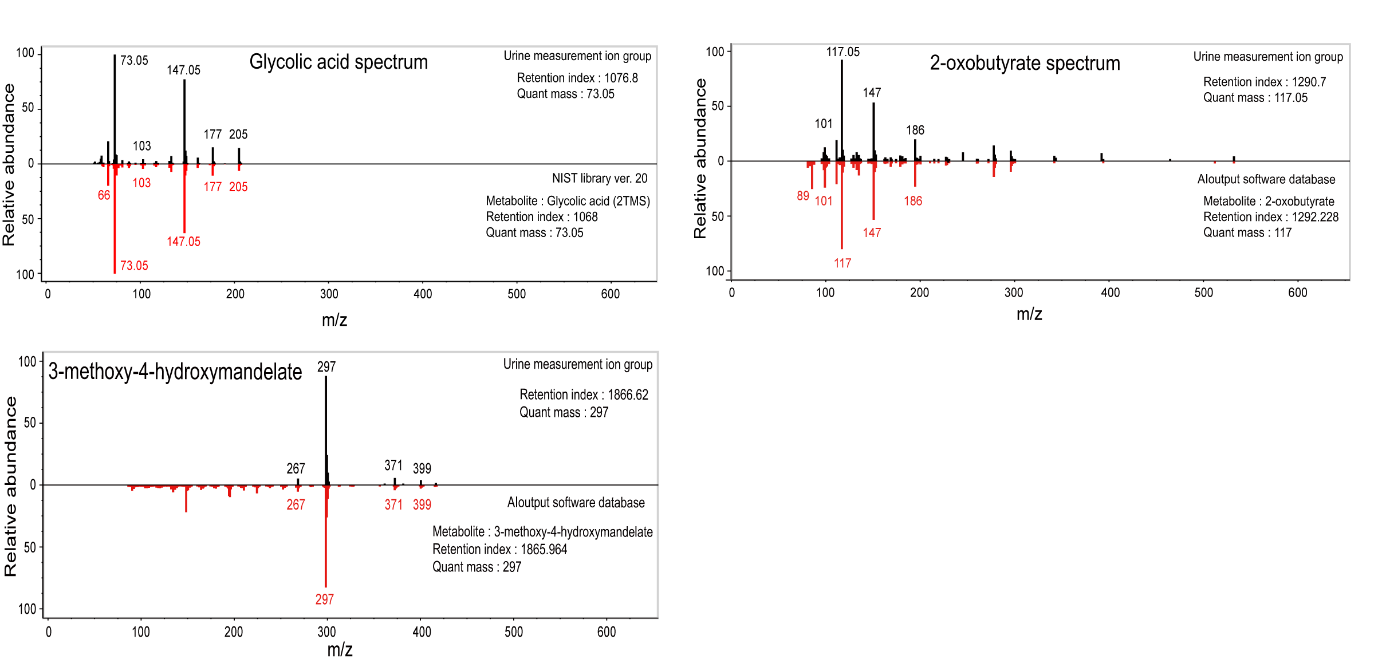


**Supplementary Figure 8.** Comparison of mass spectra of metabolites presented in Figure 2 and standard substances or gas chromatography mass spectrometry (GC-MS) libraries. The red line indicates the major mass-to-charge ratio (m/z). Alanine, leucine, isoleucine, threonine, glycine, serine, lactic acid, malonic acid, fructose, hippuric acid, mannitol, serotonin, ribose and N-acetyl-D-glucosamine were identified using standard solutions. glycolic acid was identified by comparing their spectra with those in the NIST library, whereas 2-oxobutyrate and 3-methoxy-4-hydroxymandelate were identified using AIoutput software library.
